# Supplementary material for: Cool Temperature Enhances Growth, Ferulic Acid and Flavonoid Biosynthesis While Inhibiting Polysaccharide Biosynthesis in Angelica sinensis
Source: Molecules. 2022 Jan 5;27(1):320. doi: 10.3390/molecules27010320 (PMC8746531; doi:10.3390/molecules27010320)
Supplement: Supplementary file 1 [file molecules-27-00320-s001.zip › molecules-1526119-supplementary.pdf]

Figure supplementary materials

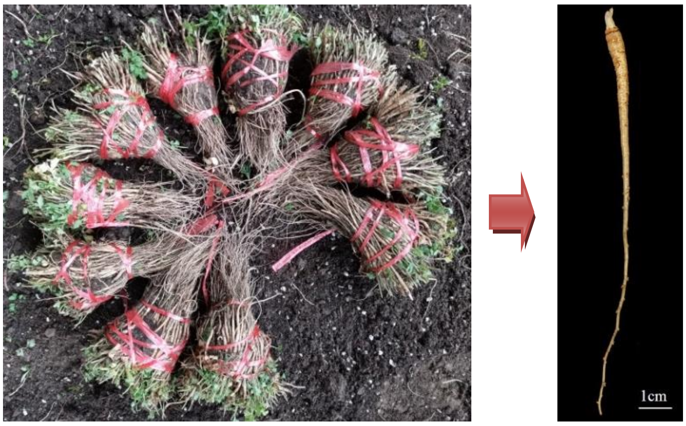

Fig. S1 Seedlings of *A. sinensis* (cultivar Mingui 1) with root-tip diameter 0.4-0.5 cm.

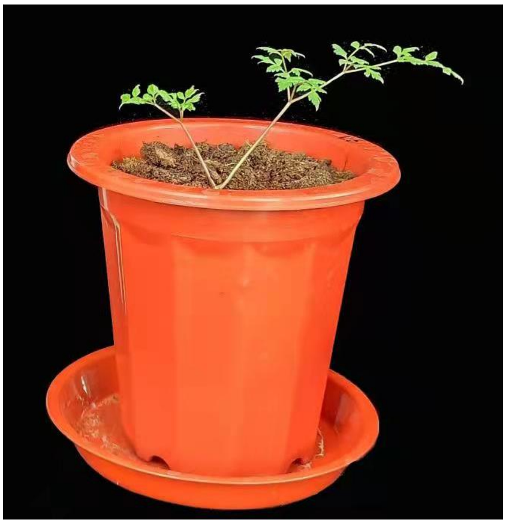

Fig. S2 Plantlets contained two leaves after 15 days.

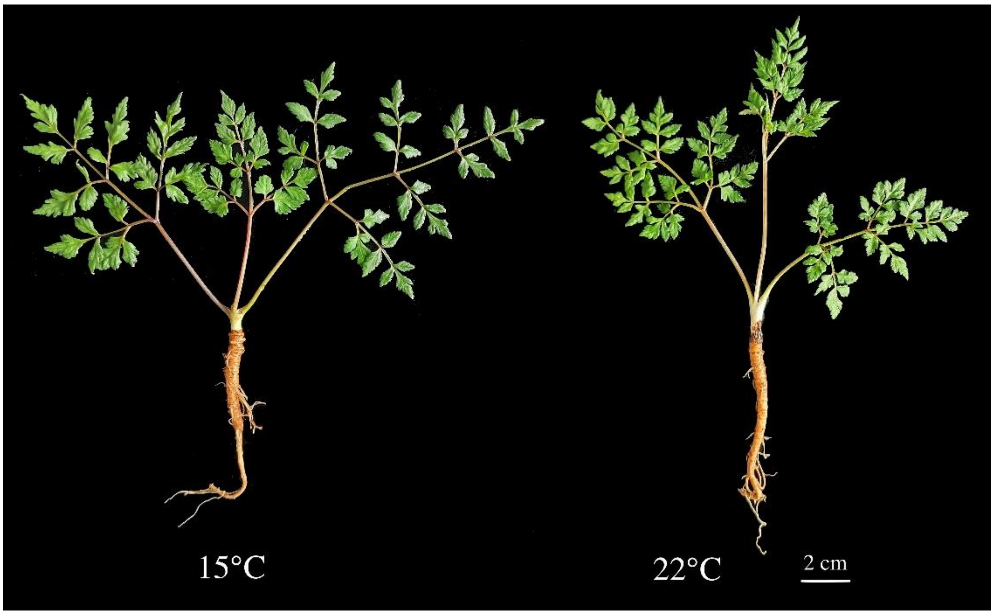

Fig. S3 Plants treated at 15 and 22°C after 30 days.

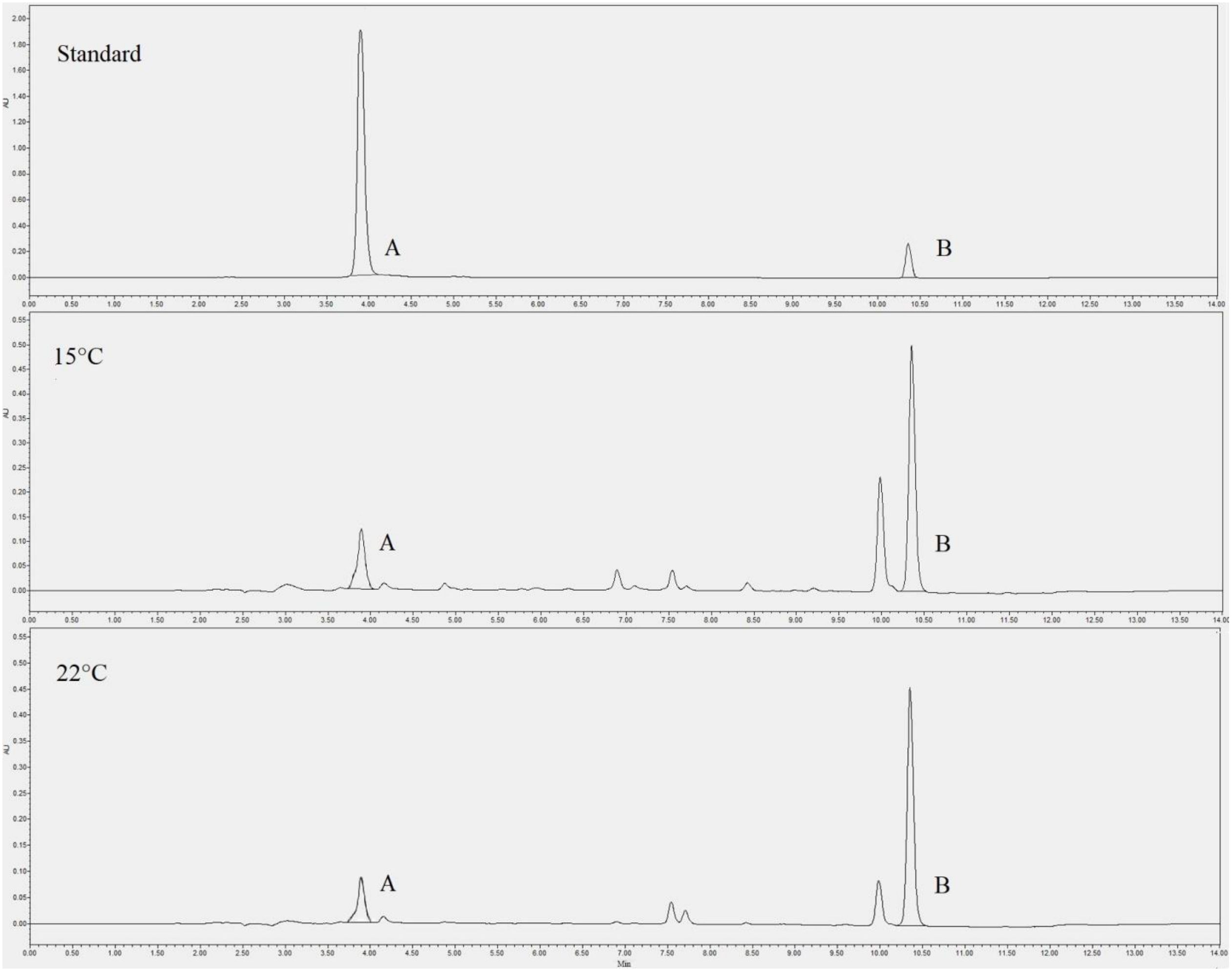

Fig. S4 Representative HPLC chromatograms of standard and samples at 15°C and 22°C. A and B represent ferulic acid and ligustilide.
